# Development and multi-database validation of machine learning models for 28-day mortality prediction in ICU patients with COPD and pulmonary hypertension

**DOI:** 10.3389/fmed.2026.1959885

**Published:** 2026-10-14

**Authors:** Yuwei Xia, Yuxuan Yang, Junchao Yang

**Affiliations:** 1 The First Affiliated Hospital of Zhejiang Chinese Medical University (Zhejiang Provincial Hospital of Chinese Medicine), Hangzhou, Zhejiang, China; 2 Pharmacy 4 Less Pty Ltd, Sydney, NSW, Australia

**Keywords:** chronic obstructive pulmonary disease, machine learning, mortality prediction, multi-database validation, pulmonary hypertension, SHAP analysis

## Abstract

**Background:**

Early mortality prediction in critically ill patients with chronic obstructive pulmonary disease (COPD) coexisting pulmonary hypertension (PH) remains challenging, particularly because prediction models developed in intensive care unit (ICU) database may not perform consistently across other healthcare systems.

**Methods:**

This retrospective multicenter study employed two large-scale databases to establish and validate ML models for predicting early mortality in ICU patients with COPD and PH. The MIMIC-IV database served as the model-development cohort (2555 patients), with external validation conducted on eICU CRD (292 patients) database. Six ML algorithms were adopted: Random Forest (RF), Extreme Gradient Boosting (XGBoost), Logistic Regression (LR), Light Gradient Boosting Machine (LightGBM), Support Vector Machines (SVM), and Artificial Neural Network (ANN).

The baseline variables were screened to identify potential predictors via least absolute shrinkage and selection operator (Lasso) regression. Model interpretability was evaluated via SHapley Additive exPlanations (SHAP) analysis.

**Results:**

The overall cohort consisted of 2,847 ICU patients with COPD and PH. Twelve consistently crucial clinical features were identified, including acute kidney injury, pneumonia, age, weight, heart rate, red blood cell distribution width (RDW), white blood cell (WBC), anion gap, chloride, creatinine, base excess, potential of hydrogen (pH). The Random Forest algorithm achieved the optimal performance, with a training Area Under Curve (AUC) of 0.938, internal validation AUC of 0.748, and eICU CRD external validation AUC of 0.800. SHAP analysis indicated that acute kidney injury was the most influential predictor, followed by base excess and RDW. Additionally, a web tool was developed in this study to facilitate the application of early death risk prediction in clinical practice.

**Conclusions:**

This study is the multi-database validation of ML models for early mortality prediction in ICU patients with COPD and PH. The Random Forest model integrated with SHAP interpretability offers a reliable tool for clinical decision-making support, with consistent validity verified across two databases, including our internal validation cohort. This work provides a decision support tool to assist in identifying high-risk individuals within this complex comorbid population, and establishes a foundation for future studies with a more dynamic and personalized prognostic approach.

## Introduction

Pulmonary hypertension (PH) is a haemodynamic condition characterized by an elevated mean pulmonary arterial pressure (mPAP ≥ 20 mmHg) at rest, which can ultimately lead to right ventricular remodelling and right heart failure (1). It has been estimated that at least 1% of the global population are affected by pulmonary hypertension, with all-cause mortality >50% within 5 years (2). Chronic obstructive pulmonary disease (COPD) is a chronic inflammatory airway disease with persistent airflow limitation, and can potentially induce chronic bronchitis, emphysema, and small airway remodeling (3). According to the Global Initiative for Chronic Obstructive Lung Disease 2025 Report (GOLD 2025) (4), the global prevalence of COPD accounts for about 10.3% among adults, and the mortality rate remains high, which is currently the third leading cause of death in the world. It is noteworthy that a meta-analysis covering a wide range of COPD severity levels revealed that approximately 39.2% of COPD patients suffered from associated PH (5). Furthermore, in patients with severe COPD, this proportion can even reach 90% (6). The coexistence of COPD and PH significantly deteriorates patient clinical status and reduces long-term survival rates (7). These interrelated epidemiological and clinical characteristics emphasize the need of early risk identification for patients with COPD and PH. Accurately predicting patient outcomes is essential to promote timely optimization of treatment regimens.

Machine learning (ML) techniques have emerged as a powerful tool for medical prediction, due to their function to automatically extract features from high-dimensional datasets and capture intricate non-linear correlations between clinical variables (8). Compared to traditional prediction models, ML-based approaches demonstrated superior performance in disease prediction and prognosis in cardiopulmonary disease research (9, 10). However, there is currently no ML prediction model developed and validated for COPD and PH.

To address this gap, this study constructed and validated a machine learning prediction model for early mortality in COPD and PH patients utilizing the data from two large databases. In addition, by integrating SHapley Additive exPlanations (SHAP) analysis, this study provides visual clinical insights, that may support clinical decision-making and early intervention of this high-risk population.

## Methods

### Data source

All the clinical data of the patients with COPD and PH in this study were obtained from the source: the MIMIC-IV v3.1 database, and eICU v2.0 database. The MIMIC-IV and eICU-CRD data were accessed for research purposes on 15 March 2026 and 26 March 2026, respectively. The authors had access only to de-identified data and did not have access to information that could identify individual participants during or after data collection. One author completed the Collaborative Institutional Training Initiative (CITI) Program (Record ID 73874649), obtained access to these restricted-access databases. The original data collection in each source database was approved by the institutional review board, and informed consent was obtained from all participants. This study was conducted in accordance with the Declaration of Helsinki.

### Database descriptions

#### MIMIC-IV database

Medical Information Mart for Intensive Care IV (MIMIC-IV) database version 3.1 contains de-identified health records from patients admitted to the ICU at Beth Israel Deaconess Medical Center between 2008 and 2019, and it served as the primary training dataset for model development (11).

#### eICU database

The eICU Collaborative Research Database (eICU CRD) version 2.0 contains data from 335 ICUs across the United States between 2014 and 2015 and was used for external validation (12).

### Participants

This study used the International Classification of Diseases, Ninth Revision, Clinical Modification (ICD-9-CM) and the International Classification of Diseases, Tenth Revision, Clinical Modification (ICD-10-CM) diagnostic codes to operationally identify and enroll patients with COPD and PH. PH was identified using ICD-9-CM code 4160 and ICD-10-CM codes I270, I272, I2720–I2724, and I2729. COPD was identified using ICD-9-CM codes 490, 496, 4910, 4911, 4918–4920, 4928, 4940, 4941, 49120–49122 and ICD-10-CM codes J40, J42, J44, J410, J411, J430–J432, J438–J441, and J449. The core code sets were consistent with previously published MIMIC-IV studies of COPD and PH (13, 14).

Patients diagnosed with PH according to the International Classification of Diseases, 9th Revision (ICD-9: 4160) and 10th Revision (ICD-10: I270, I272, I2720-I2724, I2729), and diagnosed with COPD according to the International Classification of Diseases, 9th Revision (ICD-9: 490, 496, 4910, 4911, 4918–4920, 4928, 4940, 4941, 49120–49122) and 10th Revision (ICD-10: J40, J42, J44, J410, J411, J430-J432, J438-J441, J449) were included in the study.

The inclusion criteria were: (1) patients diagnosed with COPD and PH based on ICD codes; (2) age between 18 and 100 years; (3) length of ICU stay ≥ 24 h; (4) first ICU admission. Patients with more than 20% missing essential clinical data were excluded from the analysis.

### Variable extraction

The following information of the patients from the MIMIC-IV, and eICU CRD databases was used: (1) demographic characteristics (age, gender, weight, race, language, and marital status), (2) vital signs (heart rate, respiratory rate, temperature, systolic blood pressure (SBP), diastolic blood pressure (DBP), mean blood pressure (MBP), oxygen saturation (SpO_2_)), (3) laboratory tests (hematocrit, hemoglobin, red cell distribution width (RDW), red blood cells (RBC), white blood cells (WBC), platelet count, anion gap, calcium, potassium, sodium, chloride, glucose, creatinine, blood urea nitrogen (BUN), potential of hydrogen (pH), PCO_2_, Base excess, international normalized ratio (INR), prothrombin time (PT), partial thromboplastin time (PTT)), (4) comorbidities (hypertension, acute kidney injury, cirrhosis, hepatitis, pulmonary tuberculosis, pneumonia, cerebrovascular accident, chronic kidney disease, malignant tumor, diabetes, hyperlipidemia, chronic bronchitis, heart failure, myocardial infarction, ischemic heart disease). Data on vital signs and laboratory tests were based on the first examination within 24 h in ICU.

### Data preprocessing

Variables with more than 20% missingness were first excluded, followed by patients with missing values in more than 20% of the retained variables. The MIMIC-IV cohort was then randomly divided into a training set (70%) and an internal validation set (30%) using stratified sampling implemented with the “createDataPartition” function in the R package “caret”, thereby preserving the outcome distribution across the two subsets. Missing data were subsequently handled using chained equations implemented with the “mice” package in R. The training and internal validation datasets were imputed separately, and all feature selection procedures and model construction processes were performed exclusively within the imputed training dataset.

### Feature selection

Candidate baseline predictors were selected exclusively within the training set using least absolute shrinkage and selection operator (LASSO) logistic regression. Lasso is a linear regression-based model that is regularized by imposing an L1 penalty on the regression coefficients. To find the optimal constraint parameter, LASSO regression with 10-fold cross-validation was used to screen variables. LASSO feature selection was not repeated within each model-tuning fold. Neither the internal validation set nor the external validation cohort was involved in the feature-selection procedure.

### Model establishment and comparison

Six machine learning algorithms were developed using Python 3.10: Random Forest (RF), Extreme Gradient Boosting (XGBoost), Logistic Regression (LR), Light Gradient Boosting Machine (LightGBM), Support Vector Machines (SVM), and Artificial Neural Network (ANN). To ensure model stability, 5-fold cross-validation and grid search were employed for hyperparameter optimization, and select the optimal model based on the Area Under Curve (AUC) value of the internal validation set. The performance of the models was compared based on multiple indicators, including the area under the receiver operating characteristic curve (ROC-AUC), accuracy, sensitivity, specificity, F1 score, and confusion matrix. The ROC curves of the training and the validation sets were compared, and the calibration curve and decision curve analysis (DCA) were performed on the validation set to evaluate the clinical application value of each model.

### Model interpretation

The Shapley Additive Explanations (SHAP) method was employed to to investigate the contribution of each feature variable to the final output of the model. SHAP analysis utilizes game theory methods to compensate for the black box nature of high-performance machine learning models, focuses on the blind spots of complex information, and significantly enhances the interpretability of the model (15).

### Statistical analysis

R software (version 4.5.1) was utilized for statistical analysis. Initially, the baseline statistical values of all variables were calculated and the normality of continuous variables were assessed using the Shapiro–Wilk test. For measurement data following a normal distribution, t-tests were employed; for those not following a normal distribution, Wilcoxon rank-sum tests were used. Categorical variables were compared using the *χ^2^* test. In statistical results, a *p* value < 0.05 indicates a statistically significant difference.

## Results

### Baseline characteristics

The patient selection process for the database is illustrated in Figure 1. After excluding patients who were not admitted to the ICU for the first time, were younger than 18 years old or older than 100 years old, had an ICU stay of less than 24 h, and those with more than 20% missing individual data, a total of 2847 patients with COPD and PH were ultimately included: 1,790 from MIMIC-IV (training set), 765 from MIMIC-IV (internal validation set), and 292 from eICU CRD (external validation set). The outcome of this model focuses on early mortality (28-day mortality rate) in COPD and PH. In MIMIC-IV training set, 1,372 patients survived and 418 patients died. Baseline analysis primarily focuses on the outcome of 28-day mortality rate (Table 1).

**Figure 1:**
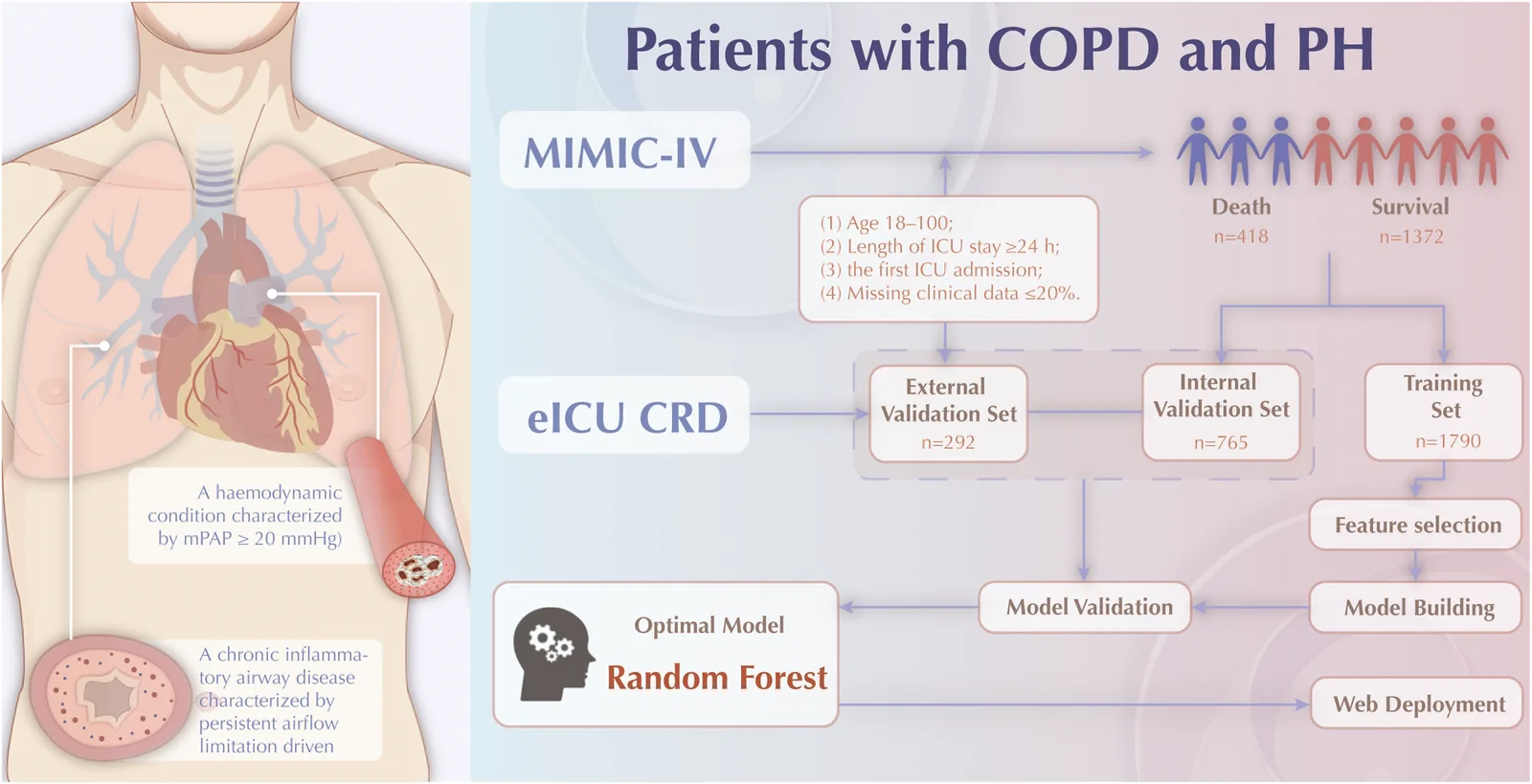
Workflow diagram. MIMIC-IV, Medical Information Mart for Intensive Care IV; eICU CRD, eICU Collaborative Research Database.

**Table 1:** Baseline table.

**Variables**	**Overall**	**Survival**	**Death**	***P* value**
Number (sample size)	1,790	1,372	418	
Gender n(%)				0.534
Male	891 (49.78)	689 (50.22)	202 (48.33)	
Female	899 (50.22)	683 (49.78)	216 (51.67)	
Race n(%)				0.935
Others	249 (13.91)	190 (13.85)	59 (14.11)	
White	1,283 (71.68)	982 (71.57)	301 (72.01)	
Black	258 (14.41)	200 (14.58)	58 (13.88)	
Language n(%)				0.615
Others	179 (10.00)	134 (9.77)	45 (10.77)	
English	1,611 (90.00)	1,238 (90.23)	373 (89.23)	
Marital status n(%)				0.845
Others	415 (23.18)	314 (22.89)	101 (24.16)	
Married	857 (47.88)	661 (48.18)	196 (46.89)	
Single	518 (28.94)	397 (28.94)	121 (28.95)	
Hypertension n(%)				0.032
NO	1,417 (79.16)	1,070 (77.99)	347 (83.01)	
YES	373 (20.84)	302 (22.01)	71 (16.99)	
Acute kidney injury n(%)				<0.001
NO	871 (48.66)	739 (53.86)	132 (31.58)	
YES	919 (51.34)	633 (46.14)	286 (68.42)	
Cirrhosis n(%)				0.014
NO	1,661 (92.79)	1,285 (93.66)	376 (89.95)	
YES	129 (7.21)	87 (6.34)	42 (10.05)	
Hepatitis n(%)				0.006
NO	1,701 (95.03)	1,315 (95.85)	386 (92.34)	
YES	89 (4.97)	57 (4.15)	32 (7.66)	
Pulmonary tuberculosis n(%)				1.000
NO	1,787 (99.83)	1,370 (99.85)	417 (99.76)	
YES	3 (0.17)	2 (0.15)	1 (0.24)	
Pneumonia n(%)				<0.001
NO	1,276 (71.28)	1,018 (74.20)	258 (61.72)	
YES	514 (28.72)	354 (25.80)	160 (38.28)	
Cerebrovascular accident n(%)				0.027
NO	1,618 (90.39)	1,228 (89.50)	390 (93.30)	
YES	172 (9.61)	144 (10.50)	28 (6.70)	
Chronic kidney disease n(%)				0.018
NO	1,151 (64.30)	903 (65.82)	248 (59.33)	
YES	639 (35.70)	469 (34.18)	170 (40.67)	
Malignt tumor n(%)				0.872
NO	1,505 (84.08)	1,152 (83.97)	353 (84.45)	
YES	285 (15.92)	220 (16.03)	65 (15.55)	
Diabetes n(%)				0.594
NO	1,148 (64.13)	885 (64.50)	263 (62.92)	
YES	642 (35.87)	487 (35.50)	155 (37.08)	
Hyperlipidemia n(%)				0.008
NO	894 (49.94)	661 (48.18)	233 (55.74)	
YES	896 (50.06)	711 (51.82)	185 (44.26)	
Chronic bronchitis n(%)				0.063
NO	1,381 (77.15)	1,073 (78.21)	308 (73.68)	
YES	409 (22.85)	299 (21.79)	110 (26.32)	
Heart failure n(%)				<0.001
NO	557 (31.12)	457 (33.31)	100 (23.92)	
YES	1,233 (68.88)	915 (66.69)	318 (76.08)	
Myocardial infarction n(%)				0.008
NO	1,491 (83.30)	1,161 (84.62)	330 (78.95)	
YES	299 (16.70)	211 (15.38)	88 (21.05)	
Ischemic heart disease n(%)				0.443
NO	888 (49.61)	688 (50.15)	200 (47.85)	
YES	902 (50.39)	684 (49.85)	218 (52.15)	
Age (years, median [IQR])	73.00[63.00, 82.00]	73.00[63.00, 81.00]	75.00[65.00, 84.00]	0.004
Weight (kg, median [IQR])	79.25[66.00, 96.57]	80.60[66.80, 99.03]	74.15[63.65, 90.95]	<0.001
Heart rate (times/minute, median [IQR])	86.00[75.00, 100.00]	85.00[74.00, 99.00]	91.00[76.00, 106.75]	<0.001
Respiratory rate (times/minute, median [IQR])	19.00[16.00, 24.00]	19.00[15.00, 24.00]	20.00[17.00, 25.00]	<0.001
SPO2 (%, median [IQR])	97.00[94.00, 100.00]	97.00[94.00, 100.00]	97.00[93.00, 100.00]	0.108
SBP (mmHg, median [IQR])	116.00[102.00, 134.00]	116.00[102.00, 134.00]	114.00[98.25, 131.00]	0.020
DBP (mmHg, median [IQR])	67.00[56.00, 79.75]	67.00[57.00, 80.00]	66.00[54.25, 78.75]	0.437
MBP (mmHg, median [IQR])	80.00[69.00, 92.75]	80.00[70.00, 92.25]	79.00[69.00, 92.75]	0.247
Temperature (°F, median [IQR])	98.10[97.70, 98.60]	98.20[97.70, 98.60]	98.00[97.60, 98.57]	<0.001
Hematocrit (%, median [IQR])	30.90[26.40, 36.40]	31.15[26.60, 36.50]	29.95[25.80, 35.50]	0.038
Hemoglobin (g/dL, median [IQR])	9.80[8.30, 11.60]	10.00[8.38,11.70]	9.50[8.10, 11.10]	0.006
Platelet count (10^9^/L, median [IQR])	178.00[127.00, 239.00]	176.00[127.00, 237.00]	181.00[130.25, 247.00]	0.673
RDW (%, median [IQR])	15.50[14.10, 17.40]	15.20[13.90, 17.10]	16.30[14.80, 18.30]	<0.001
RBC (10^12^/L, median [IQR])	3.37[2.85, 3.99]	3.41[2.87, 4.01]	3.26[2.76, 3.89]	0.004
WBC (10^9^/L, median [IQR])	10.70[7.60, 14.90]	10.50[7.50, 14.40]	11.00[7.80, 16.80]	0.005
Anion gap (mEq/L, median [IQR])	14.00[11.00, 17.00]	14.00[11.00, 17.00]	15.00[12.00, 19.00]	<0.001
Calcium total (mEq/L, median [IQR])	8.50[8.10, 9.00]	8.50[8.10, 9.00]	8.60[8.00, 9.00]	0.916
Chloride (mEq/L, median [IQR])	102.00[97.00, 106.00]	102.00[97.00, 106.00]	99.00[95.00, 104.00]	<0.001
Glucose (mg/dL, median [IQR])	127.00[105.00, 162.00]	126.00[105.00, 158.00]	132.00[108.00, 177.00]	0.004
Potassium (mEq/L, median [IQR])	4.30[3.90, 4.70]	4.30[3.90, 4.70]	4.30[3.90, 4.88]	0.202
Sodium (mEq/L, median [IQR])	138.00[135.00, 141.00]	138.00[135.00, 141.00]	138.00[134.00, 141.00]	0.151
Creatinine (mg/dL, median [IQR])	1.20[0.80, 2.00]	1.10[0.80, 1.80]	1.50[1.00, 2.40]	<0.001
BUN (mg/dL, median [IQR])	26.00[16.00, 44.00]	23.00[15.00, 41.00]	34.00[23.00, 52.00]	<0.001
Base excess (mmol/L**,** median [IQR])	0.00[−2.60, 3.00]	0.00[−2.00, 3.00]	−1.00[−5.00, 3.00]	<0.001
PCO2 (mmHg, median [IQR])	43.40[38.00, 51.00]	43.30[38.00, 51.00]	44.00[37.00, 53.15]	0.903
pH (median [IQR])	7.37[7.32, 7.42]	7.38[7.33, 7.42]	7.35[7.29, 7.40]	<0.001
INR (median [IQR])	1.40[1.20, 1.73]	1.40[1.20, 1.70]	1.50[1.20, 1.90]	<0.001
PT (seconds, median [IQR])	15.40[13.10, 19.10]	15.20[13.00, 18.50]	16.10[13.30, 21.25]	<0.001
PTT (seconds, median [IQR])	32.00[28.00, 40.00]	31.70[28.00, 39.10]	33.15[28.80, 43.80]	0.005

SPO2, Oxygen Saturation; SBP, Systolic Blood Pressure; DBP, Diastolic Blood Pressure; MBP, Mean Blood Pressure; RDW, Red Cell Distribution Width; RBC, Red Blood Cells; WBC, White Blood Cells; BUN, Blood Urea Nitrogen; pH, potential of hydrogen; INR, International Normalized Ratio; PT, Prothrombin Time; PTT, Partial Thromboplastin Time.

### Feature selection

Using the Lasso algorithm and 10-fold cross-validation in the training set, a *λ* value (*λ*.1se) of 0.026 was obtained under 1 standard error, and twelve variables were screened as predictors for establishing the machine learning prediction models at this value (Figures 2A,B). These variables included acute kidney injury, pneumonia, age, weight, heart rate, RDW, WBC, anion gap, chloride, creatinine, base excess, pH.

**Figure 2:**
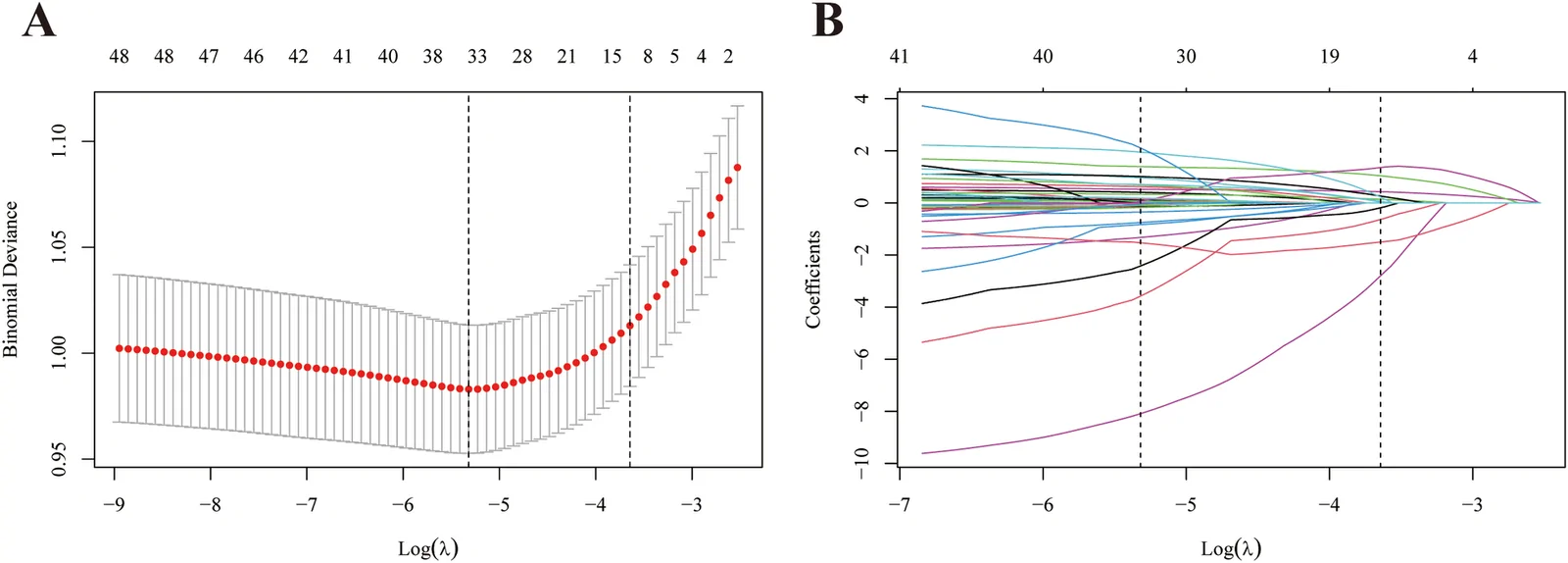
Feature selection results of lasso regression. **(A)** Optimal *λ* selection in Lasso regression using 10-fold cross-validation. The misclassification error for different variables with respect to log(*λ*) is shown. The two vertical dashed lines indicate the optimal values under the minimum and 1-standard error (1-SE) criteria, respectively. **(B)** Distribution of Lasso regression coefficients for all baseline features.

### Model development and performance evaluation

Based on the 12 predictors selected using LASSO, six machine learning models were developed and the predictive performance was evaluated for each model for COPD and PH mortality. After defining the hyperparameter search range for each model, conducting grid search using GridSearchCV, and performing 5-fold cross-validation, the optimal hyperparameter combinations were obtained. Detailed results are listed in Supplementary Table S1.

To clarify the performance of each model, their various metrics were evaluated on both the training and validation sets of MIMIC-IV, including AUC, accuracy, sensitivity, specificity, F1 score, confusion matrix (Supplementary Figure S1), Receiver Operating Characteristic (ROC), calibration curve, and Decision Curve Analysis (DCA). The performance of each model in the training set is detailed in Supplementary Table S2 and Supplementary Figure S2.

Random Forest is the optimal model based on the AUC performance of the internal validation set (Table 2). In the MIMIC-IV training set, Random Forest achieved an excellent AUC of 0.938 (95% CI: 0.925 and 0.949) (Supplementary Table S2). In the MIMIC-IV validation set (Figures 3A,B), and eICU CRD (Figure 4B and Supplementary Table S3) datasets, the AUC of Random Forest were 0.748 (95% CI: 0.711 and 0.783), and 0.800 (95% CI: 0.728 and 0.865) respectively, demonstrating good internal and external validation performance.

**Table 2:** Performance of models in internal validation set.

Models	AUC	AUC 95% CI Lower	AUC 95% CI Upper	Accuracy	Sensitivity	Specificity	F1 Score
LR	0.744	0.701	0.781	0.725	0.612	0.760	0.509
RF	0.748	0.711	0.783	0.688	0.691	0.687	0.507
XGBoost	0.740	0.703	0.777	0.656	0.742	0.630	0.501
LightGBM	0.732	0.693	0.769	0.610	0.860	0.535	0.507
SVM	0.737	0.697	0.776	0.631	0.815	0.576	0.507
ANN	0.724	0.680	0.765	0.675	0.697	0.668	0.499

**Figure 3:**
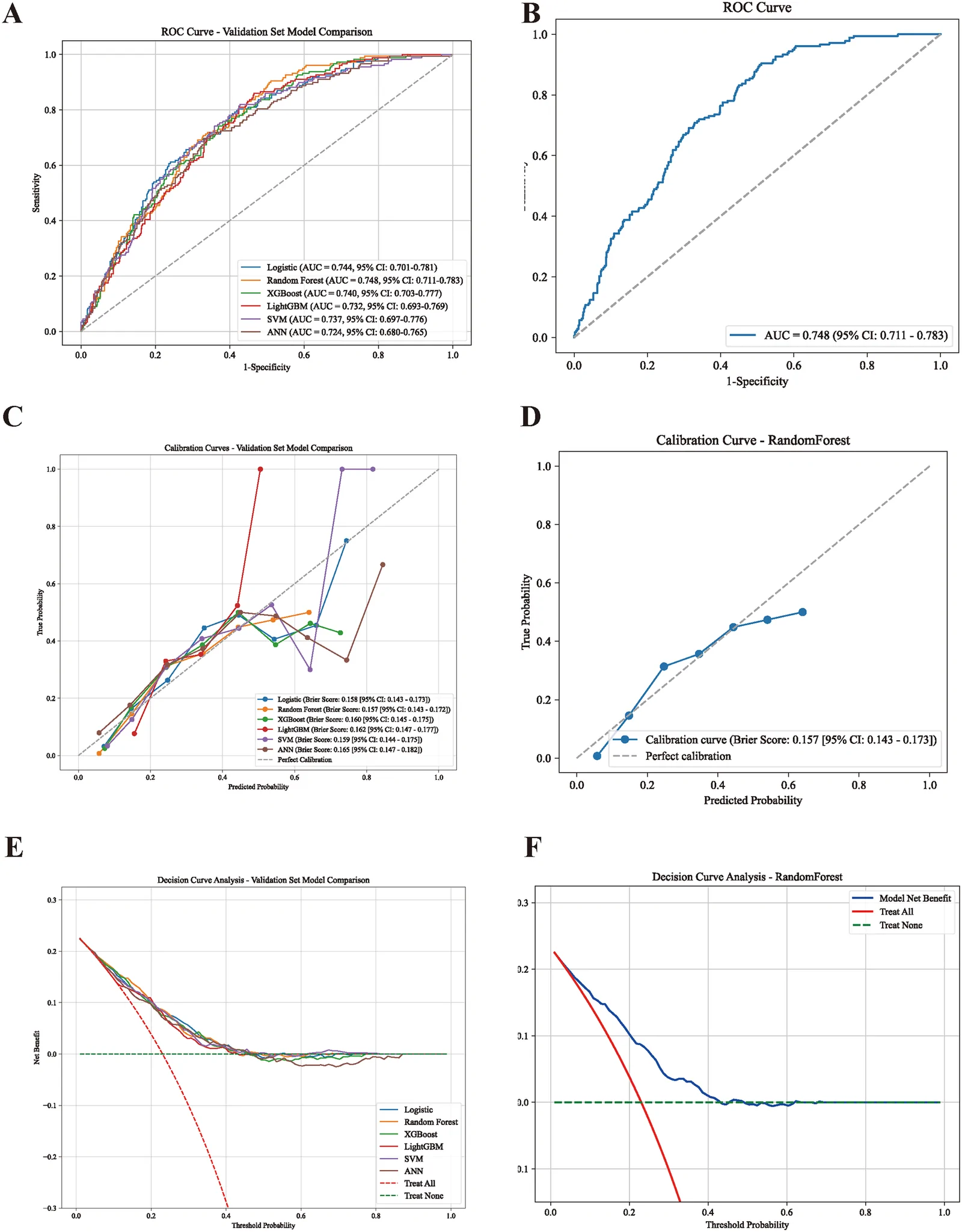
ROC curves, calibration curves, and DCA curves of various models based on the internal validation set. **(A)** Comparison of ROC curves of 6 different machine learning models. **(B)** ROC curve of Random Forest model. **(C)** Comparison of calibration curves of 6 different machine learning models. **(D)** calibration curve of Random Forest model. **(E)** Comparison of DCA curves of 6 different machine learning models. **(F)** DCA curve of Random Forest model.

**Figure 4:**
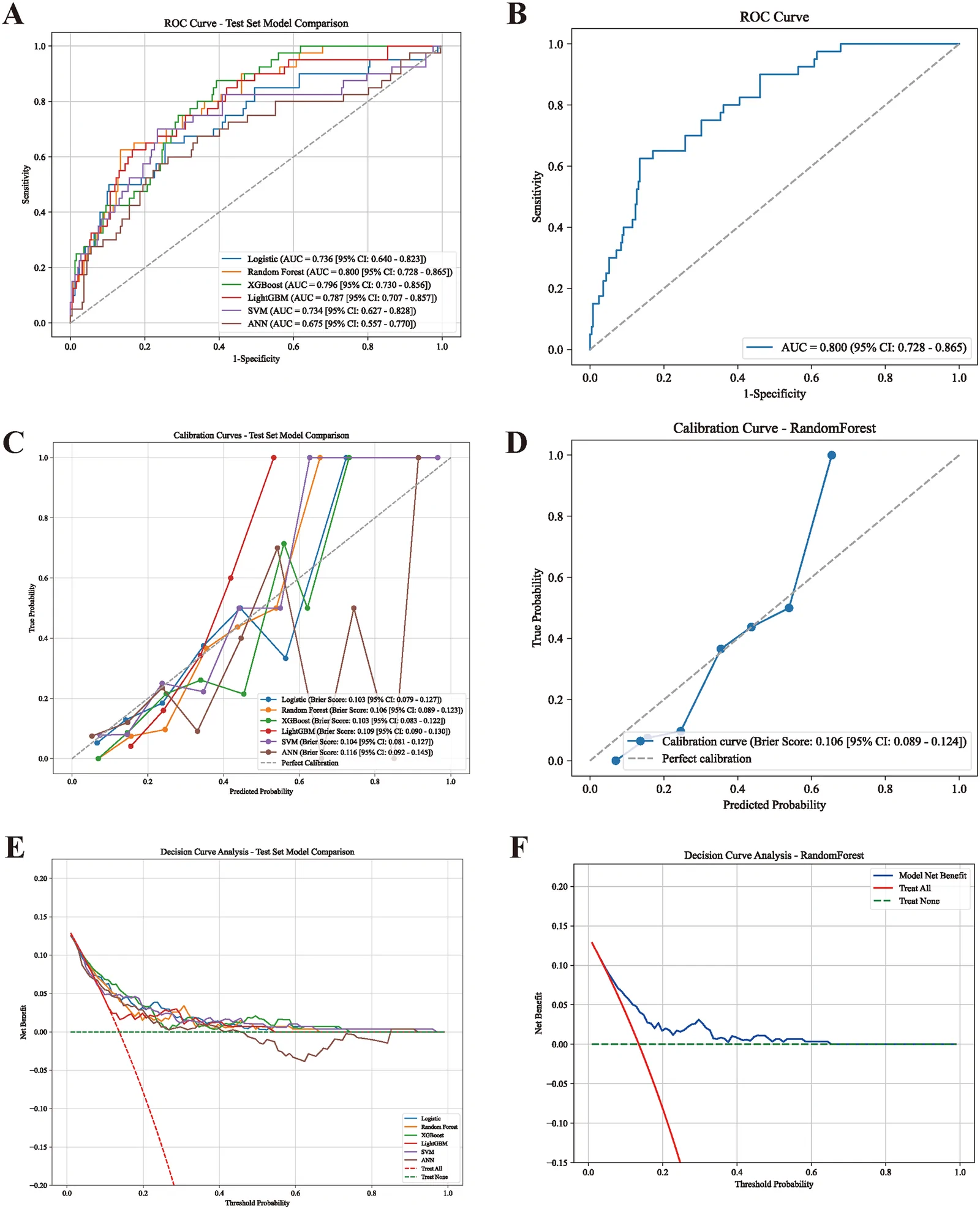
ROC curve, calibration curve, and DCA curve of the random forest model based on the eICU CRD external validation set. **(A)** Comparison of ROC curves of 6 different machine learning models. **(B)** ROC curve of Random Forest model. **(C)** Comparison of calibration curves of 6 different machine learning models. **(D)** calibration curve of Random Forest model. **(E)** Comparison of DCA curves of 6 different machine learning models. **(F)** DCA curve of Random Forest model.

The calibration curves display the Brier scores of each model. The deviations between the model outputs and the actual values are all within acceptable ranges, with Brier scores ranging from 0.113∼0.159 (Supplementary Figure S2) and from 0.157∼0.165 (Figures 3C,D) in the MIMIC-IV training and validation sets, respectively. In the external validation set, the Brier score of the Random Forest model was 0.106 (95% CI: 0.089–0.124) (Figure 4D).

DCA confirmed the clinical utility of the Random Forest model. In the MIMIC-IV internal validation set, the Random Forest model demonstrated superior net benefit compared to treat-all and treat-none strategies, and exhibited an appropriate risk threshold range of 0.05∼0.60 (Figures 3E,F). In the external validation set, Random Forest exhibited risk threshold range of 0.25–0.45 (Figures 4E).

### SHAP-based feature importance and dependence analysis

To enhance the interpretability of the relationship between the predictive model and clinical variables, SHAP analysis was introduced to reveal the key predictors affecting early mortality in the Random Forest model. Figure 5A shows the 12 significant feature variables ranked according to SHAP values, with the length of the bar representing their respective contributions to the model's prediction results. Among them, acute kidney injury are the most influential predictors. Figure 5B uses a color gradient to represent the positive and negative correlations between feature variables and mortality risk. Red dots are associated with increased risk, while purple dots indicate decreased risk. For each variable, more red dots appear on the side with a positive SHAP value, the greater its value and the higher its tendency towards mortality risk. Conversely, it indicates a lower mortality risk.

**Figure 5:**
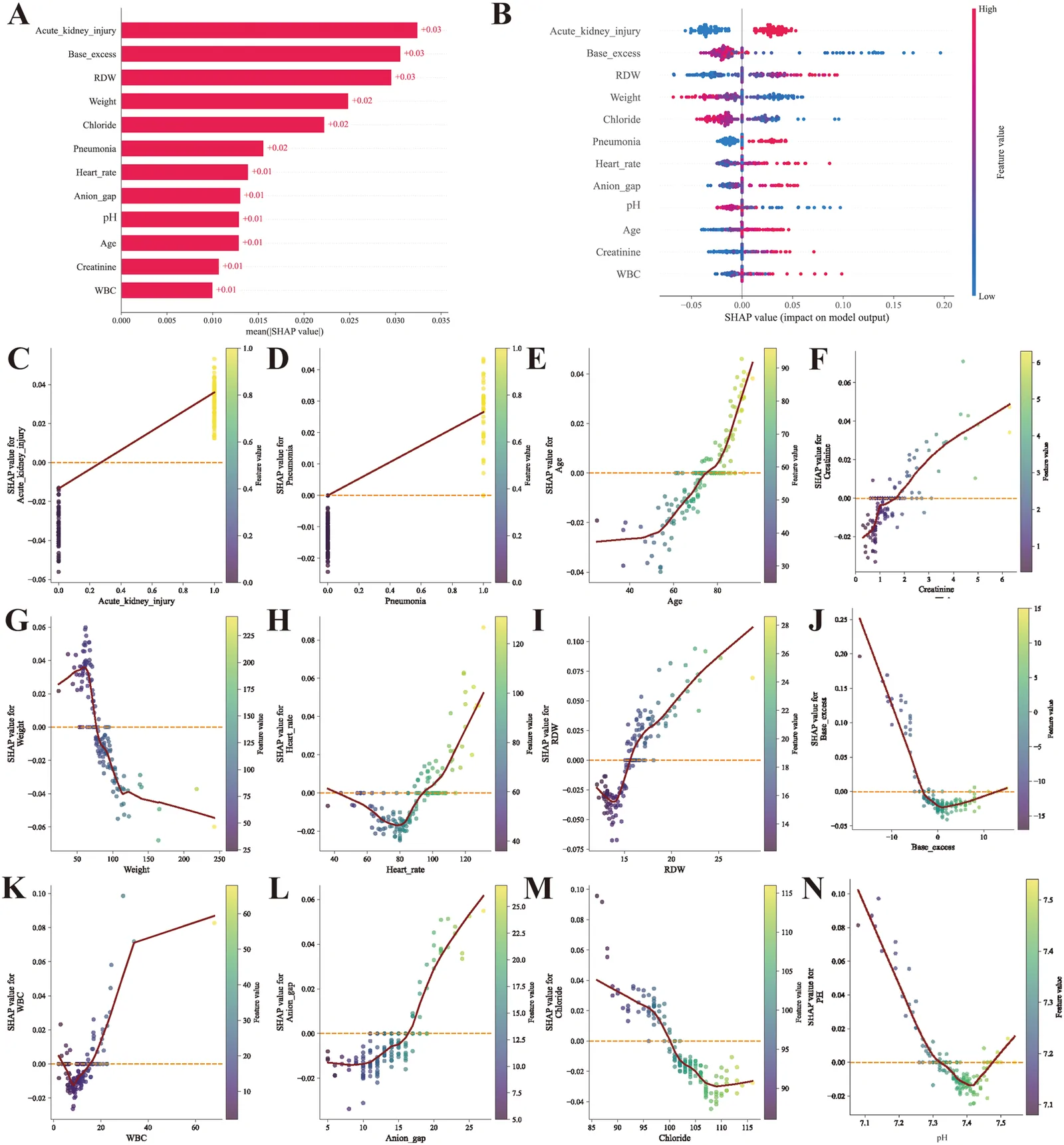
Utilize SHAP analysis to reveal the importance of each key feature variable and its correlation with mortality risk. **(A)** The 12 significant feature variables ranked according to SHAP values. **(B)** A beeswarm plot utilizing color gradients to represent the positive and negative correlations between feature variables and mortality risk. **(C–N)** Dependency graph of 12 key characteristic variables including acute kidney injury, pneumonia, age, weight, heart rate, RDW, WBC, anion gap, chloride, creatinine, base excess, and pH.

The SHAP dependence plots for each key clinical feature reveal the complex nonlinear relationships between different features and the model's predicted outcomes. The horizontal axis represents the standardized feature values, while the vertical axis represents the SHAP values, which quantify the marginal contribution strength and direction of the feature variables to the prediction results. A positive SHAP value (above the yellow line) indicates that the feature promotes the prediction, whereas a negative value indicates an inhibitory effect. The absolute value directly reflects the influence weight of the feature. When acute kidney injury, and pneumonia are “present (value=1)”, showed positive SHAP contributions, indicating that the presence of these features contributes toward a higher predicted probability. In continuous variables, the fitted lines for age, RDW, creatinine, and anion gap generally show an upward trend as the abscissa values increase. This indicates that as these variables increase, their contribution to mortality risk rises. Chloride and weight, on the other hand, exhibit the opposite trend. Heart rate, WBC, base excess, and pH first decreases and then increases, suggesting that as them increases, its contribution to mortality risk first decreases and then increases (Figures 5C–L). Furthermore, the approximate turning points for “promotion” and “inhibition” of these continuous variables are: age (75 years), weight (80 kg), heart rate (45 times/minute and 90 times/minute), RDW (15%), WBC (5 × 10^9^/L and 15 × 10^9^/L), anion gap (17 mEq/L), chloride (100 mEq/L), creatinine (1.5 mg/dL), base excess (−4 mmol/L and 15 mmol/L), and pH (7.32 and 7.48). The dependence plots provide intuitive support for analyzing the predictive value, direction of action, and potential mechanisms of each clinical feature by visualizing the correlation between standardized feature values and SHAP values. This helps to identify the core driving predictors behind the model's decision-making.

### Individual patient risk profiling

The SHAP waterfall plot and force plot visually represent the contribution differences of specific sample death risk features from the training set. In the waterfall plot, each feature variable is represented by a bar, with blue indicating a negative contribution to the prediction result, that is, reducing the risk of death. Red, on the other hand, represents the opposite, indicating an increase in the risk of death. The force plot illustrates how each feature contributes to the final prediction value f(x) starting from the baseline prediction value of the model. Blue arrows indicate a decrease in contribution, while red arrows indicate an increase in contribution.

Figures 6A,C reveal the waterfall plot and force plot of the same patient. acute kidney injury (=0), base excess (=0 mmol/L), weight (87.5 kg), RDW (14.6%), age (65 years), anion gap (=16 mEq/L), pH (=7.39), pneumonia (=0), and WBC (=14.5 × 10^9^/L) contributed toward a lower predicted probability. Conversely, chloride (97 mEq/L), heart rate (103 beats/minute), and creatinine (=1.5 mg/dL) are predictors that increase the prediction of mortality risk. Under relative effects, the final predicted value for this patient is 0.086, which is lower than the base value of 0.2332. This indicates that the patient is considered low risk.

**Figure 6:**
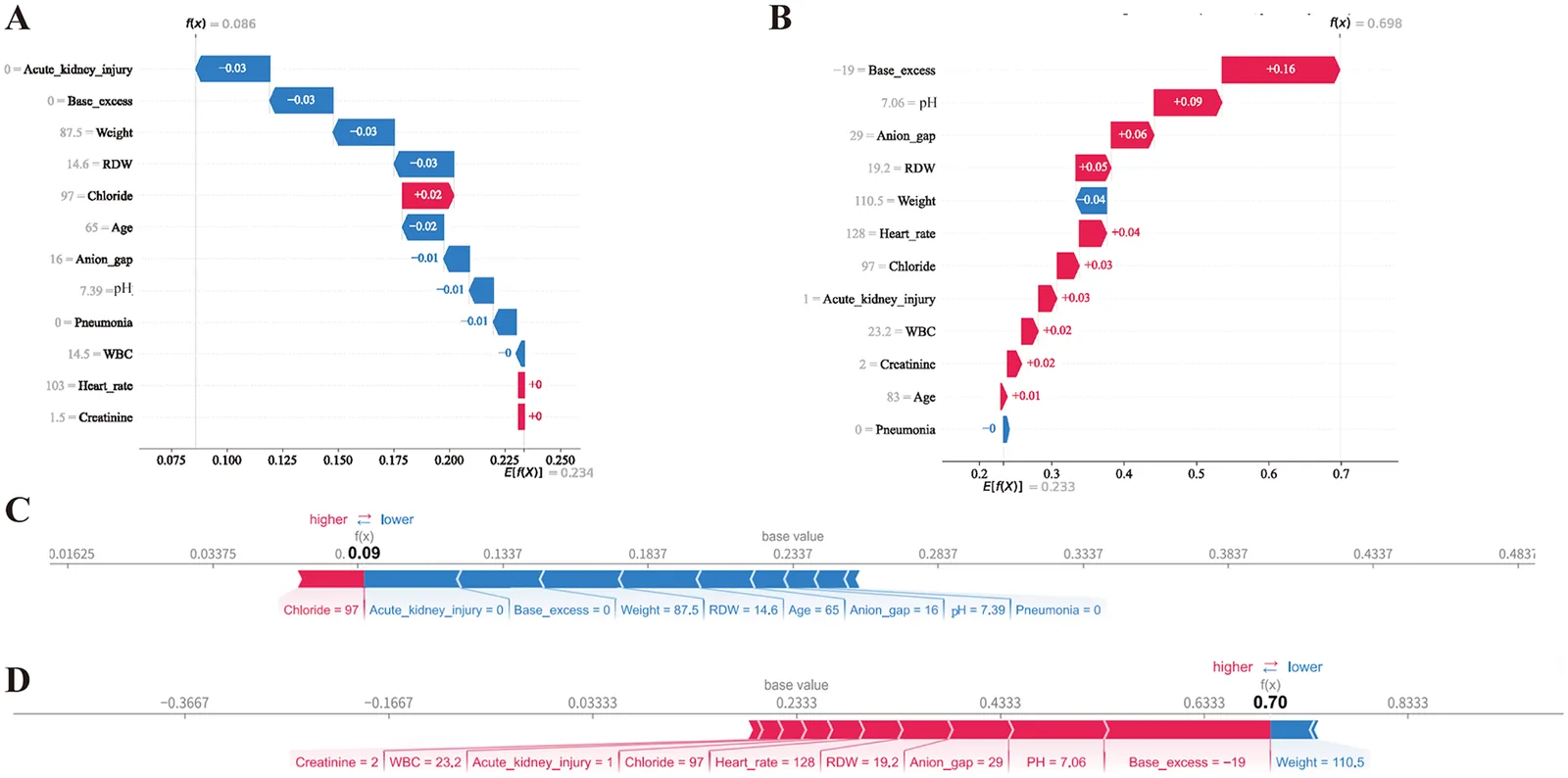
SHAP waterfall plots and force plots for COPD and PH mortality prediction. **(A)** Waterfall plot for one survival patient. **(B)** Waterfall plot for one death patient. **(C)** Force plot for one survival patient. **(D)** Force plot for one death patient.

Figures 6B,D reveal the waterfall and force plots for another patient with relatively higher risks. Base excess (=−19 mmol/L), pH (=7.06), anion gap (=29 mEq/L), RDW (19.2%), heart rate (128 beats/minute), chloride (97 mEq/L), acute kidney injury (=1), WBC (=23.1 × 10^9^/L), creatinine (=2 mg/dL), and age (83 years) are predictors that increase the prediction of mortality risk. Conversely, weight (110.5 kg), and pneumonia (=0) contribute toward a lower predicted probability. Under relative effects, the final predicted value for this patient is 0.698, which is higher than the base value of 0.2332. This indicates that the patient is considered high risk.

### Clinical application

An online prediction platform has been built using Python's Streamlit framework. Users can input demographic information, as well as vital signs, laboratory tests, medical history, etc., through the interface to achieve rapid assessment of early mortality risk in ICU patients with COPD and PH, as shown in Figure 7. Based on the SHAP interpretability analysis method, the platform displays the impact of each feature on the prediction results, including force plots and waterfall plots. Red represents that the feature increases the risk of early death, while blue indicates that it can reduce the risk. For more information about the functional description and implementation details of this platform, please refer to Supplementary Appendix S1.

**Figure 7:**
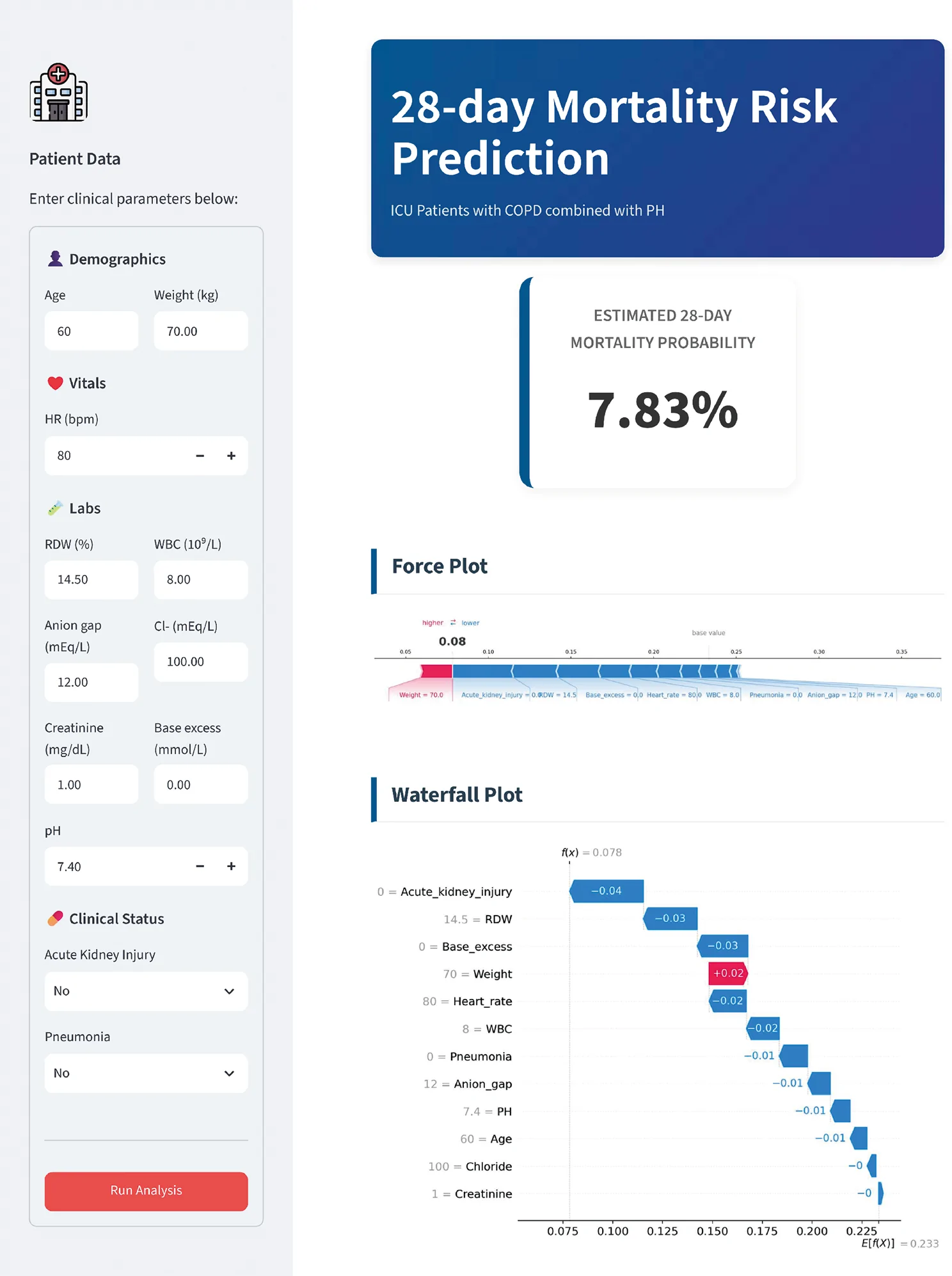
Web interface. The interface is divided into two main sections. Users can enter patient data in the sidebar on the left. The Demographics section includes age and weight. The Vitals section includes heart rate. The Labs section includes RDW, WBC, anion gap, chloride, creatinine, base excess, and pH. The Clinical status section includes acute kidney injury, and pneumonia. After filling in all the required information, users can click the “Run Analysis” button to view the risk prediction results and the contribution of each feature in the main menu on the right.

## Discussion

COPD and PH is a progressive and poor-prognosis complication of chronic respiratory diseases. A clinical study by Zeder et al. revealed that in patients with severe COPD and PH, the pulmonary vascular intima is significantly thickened, especially on the inner side (16), indicating a significant increase in pulmonary artery remodeling (17). Persistent airflow limitation complicated by pulmonary vascular remodeling causes progressive damage, leading to right heart dysfunction (18). This significantly contributes to the increased mortality rate in such comorbid patients, resulting in adverse clinical outcomes (19, 20). Therefore, clarifying the early mortality distribution characteristics and influencing predictors of this population is of great significance for improving patient prognosis. The mortality distribution in this study further verifies the high mortality risk of this population during ICU hospitalization, and highlights the need of early risk stratification, precise intervention, and further epidemiological support to reduce early mortality.

Our multicenter study utilized two large databases (MIMIC-IV, and eICU CRD), incorporating 2,847 patients in ICU across different temporal and geographical locations, to construct the machine learning prediction model for assessing early mortality in patients with COPD and PH. After systematic comparison and evaluation, the performance of Random Forest was significantly superior to other machine learning algorithms. The AUC of the training set reached 0.938, and achieving robust internal and external validation across all validation sets, with an AUC ranging from 0.748 to 0.800. Our model effectively assists clinicians in making accurate and proactive treatment decisions for patients with COPD and PH in ICU by predicting the risk of early death in such patients.

Random forest (RF), as an ensemble learning algorithm, is well suited to modeling complex nonlinear relationships and interactions among predictors without requiring prespecified functional forms. By constructing multiple decision trees from bootstrap samples and randomly selected subsets of predictors and subsequently aggregating their predictions, RF reduces the variance associated with individual decision trees and mitigates the risk of overfitting (21). Previous studies on predictive models for COPD with PH were limited and primarily focused on traditional predictive models. For instance, Zhou and colleagues conducted a study involving 535 COPD–PH patients, employing multiple logistic regression to analyze risk predictors (22). However, this study primarily emphasized long-term risk prediction and did not delve into early mortality rates among ICU patients. Additionally, traditional models may pose a potential risk of overfitting. This study illustrates the potential of combining various clinical indicators with machine learning to support early prognostic assessment in critically ill patients with COPD and PH. As emphasized by Fang et al., the integration of artificial intelligence and related technologies into the diagnosis and treatment of diseases may move beyond conventional screening and facilitate earlier and more individualized risk assessment (23).

Our study identified 12 key clinical characteristics closely associated with early patient mortality, and the impact weights of each characteristic were clarified through SHAP analysis. Acute kidney injury shows the largest contribution to the model-predicted mortality risk, which is consistent with previous studies (24). This may be because acute kidney injury induces complications such as water and sodium retention, electrolyte imbalance, and metabolic acidosis, further aggravating right ventricular overload and respiratory dysfunction, forming a vicious cycle of “lung-heart-kidney” and accelerating the deterioration of patients' conditions. Furthermore, creatinine also provides information related to renal function. Previous studies have found that higher creatinine levels are associated with increased 30-day mortality in patients with AECOPD (25), which is also consistent with our findings.

Base excess was the second most important predictor in the SHAP analysis, and pH, anion gap, and chloride were also identified as important predictors. These parameters reflect different aspects of acid–base balance and electrolyte homeostasis (26). In critically ill patients with COPD and PH, abnormalities in these parameters may arise from respiratory failure, impaired renal compensation, inadequate tissue perfusion, infection, or treatment-related fluid and electrolyte disturbances (27). Previous studies have provided support for the prognostic value of these parameters. In patients with COPD and chronic hypercapnic respiratory failure, base excess has been identified as an independent predictor of mortality (28). Notably, higher base excess in that study may primarily reflect compensatory metabolic alkalosis resulting from chronic carbon dioxide retention, whereas in the present study lower base excess was also associated with a higher predicted risk of mortality. A previous systematic review and meta-analysis further showed that acidemia is associated with short-term mortality in hospitalized patients with AECOPD, and pH is also an important prognostic indicator of mortality in ICU patients (29). Zhu et al. retrospectively analyzed PH patients with sepsis and found that a higher anion gap was associated with an increased risk of in-hospital mortality (30). Several studies have suggested that serum chloride also has potential prognostic significance. In critically ill patients with COPD, serum chloride levels showed an L-shaped relationship with 90-day and 365-day mortality, with lower chloride levels associated with a higher risk of death (31). In patients with PH, a serum chloride level ≤100 mmol/L at 6 months after diagnosis was independently associated with an increased risk of mortality (32). Overall, these results support the potential value of early acid–base status and electrolyte levels in the prognostic assessment of critically ill patients with COPD and PH, but their clinical application still requires further validation in independent prospective cohorts.

Pneumonia and WBC provided further information related to infection and inflammation. A meta-analysis of 18 studies showed that concomitant community-acquired pneumonia was associated with increased mortality and a greater need for mechanical ventilation among hospitalized patients with COPD (33). Our finding regarding WBC is also consistent with a prospective cohort study of patients with COPD, which demonstrated that a persistent systemic inflammatory phenotype, defined using a biomarker panel that included WBC, was associated with more frequent exacerbations and higher mortality (34).

Age and weight are basic demographic characteristics. Ageing is a classic risk predictor for mortality from various critical illnesses, associated with decreased physiological reserves and increased comorbidities. In this study, this association was once again confirmed. Low body weight may indicate malnutrition and muscle wasting, which are common in patients with severe COPD and are associated with immune dysfunction and poor prognosis (35).

Heart rate and RDW are indicators reflecting physiological homeostasis. An increased heart rate is often direct manifestations of severe disease, hypoxia, or infection. RDW is the third-ranking predictor. Higher RDW values are associated with inflammatory states, oxidative stress, and poor nutritional status, all of which are important components of the pathophysiological processes of COPD and PH (36, 37). The correlation of these indicators with mortality has extensive physiological support in critical care medicine.

This study also has several limitations. First, retrospective observational design cannot avoid residual confounding bias and can only establish associations rather than causation. Second, COPD and PH were operationally identified using ICD diagnosis codes, and PH could not be systematically verified or classified using echocardiographic or right-heart catheterization data. Therefore, potential diagnostic and PH-subtype misclassification cannot be excluded, and our findings should be interpreted as applying to patients with code-defined COPD and coexisting PH. Third, because laboratory and vital-sign predictors were summarized over the first 24 h rather than measured strictly at ICU admission, they may partially reflect early resuscitation and other clinical management delivered. Finally, although we included multiple variables from the databases, some predictors such as pulmonary artery pressure, echocardiographic indicators of right heart function, and patient functional status (e.g., 6-minute walk distance) were missing from the clinical databases used, which may limit the comprehensiveness of the model.

## Conclusion

This study is the comprehensive multi-database validation of ML models for early mortality prediction in ICU patients with COPD and PH. The Random Forest model integrated with SHAP interpretability offers a reliable tool for clinical decision-making support, with consistent validity verified across two databases, including our internal validation cohort. This work provides a decision support tool to assist identifying high-risk individuals within this complex comorbid population, and establishes a foundation for future studies with more dynamic and personalized prognostic approach.

## Data Availability

The datasets presented in this study can be found in online repositories. The names of the repository/repositories and accession number(s) can be found in the article/Supplementary Material.
