# Supplementary material for: Development and multi-database validation of machine learning models for 28-day mortality prediction in ICU patients with COPD and pulmonary hypertension
Source: Front Med (Lausanne). 2026 Oct 14;13:1959885. doi: 10.3389/fmed.2026.1959885 (PMC13713754; doi:10.3389/fmed.2026.1959885)
Supplement: Supplementary file 1 [file Datasheet1.pdf]

Supplementary Material

1. Table S1. Optimal hyperparameter combinations for machine learning models. - *Page 1*.

2. Figure S1. Confusion matrix of internal and external validation sets. - *Pages 2*.

3. Table S2. Performance of models in training set. - *Pages 3*.

4. Figure S2. Training set confusion matrix and model evaluation. - *Pages 4*.

5. Table S3. Performance of models in external validation set. - *Pages 5*.

6. Appendix S1. 28-day mortality rate of external validation set. - *Pages 6*.

7. Appendix S2. Platform Description. – *Pages 7*.

8. Table S4. TRIPOD reporting checklist. – *Pages 8-14*.

9. Table S5. Calibration of the external validation set. – *Pages 15*.

Table S1.

Hyperparameter search spaces and optimal combinations for the machine learning models.

Model	Hyperparameter search space	Optimal hyperparameter combination
**RF**	n_estimators: [80, 100, 150]	n_estimators: 150
	max_depth: [5, 8, 10]	max_depth: 10
	min_samples_split: [5, 8, 10]	min_samples_split: 5
	min_samples_leaf: [4, 6, 8]	min_samples_leaf: 8
**XGBoost**	learning_rate: [0.01, 0.05, 0.1]	learning_rate: 0.05
	max_depth: [3, 4, 6]	max_depth: 3
	n_estimators: [80, 100, 150]	n_estimators: 100
	subsample: [0.7, 0.8, 0.9]	subsample: 0.7
	reg_alpha: [0.5, 1, 2]	reg_alpha: 1
**LightGBM**	learning_rate: [0.01, 0.05, 0.1]	learning_rate: 0.01
	num_leaves: [15, 20, 25]	num_leaves: 25
	n_estimators: [80, 100, 150]	n_estimators: 100
	subsample: [0.7, 0.8, 0.9]	subsample: 0.7
	colsample_bytree: [0.7, 0.8, 0.9]	colsample_bytree: 0.7
	reg_alpha: [0.1, 0.5, 1, 2]	reg_alpha: 0.1
**SVM**	C: [0.01, 0.1, 1, 10]	C: 0.1
	kernel: [linear, rbf]	kernel: linear
	gamma: [scale, auto, 0.001, 0.01, 0.1]	gamma: scale
**ANN**	hidden_layer_sizes: [(25,)]	hidden_layer_sizes: (25,)
	activation: [relu, tanh]	activation: relu
	alpha: [0.001, 0.01, 0.1]	alpha: 0.1
	learning_rate_init: [0.001, 0.01, 0.1]	learning_rate_init: 0.01
	max_iter: [200, 300]	max_iter: 200

RF, Random Forest; XGBoost, Extreme Gradient Boosting; LightGBM, Light Gradient Boosting Machine; SVM, Support Vector Machines; ANN, Artificial Neural Network.

Figure S1.

Confusion matrix of internal and external validation sets.

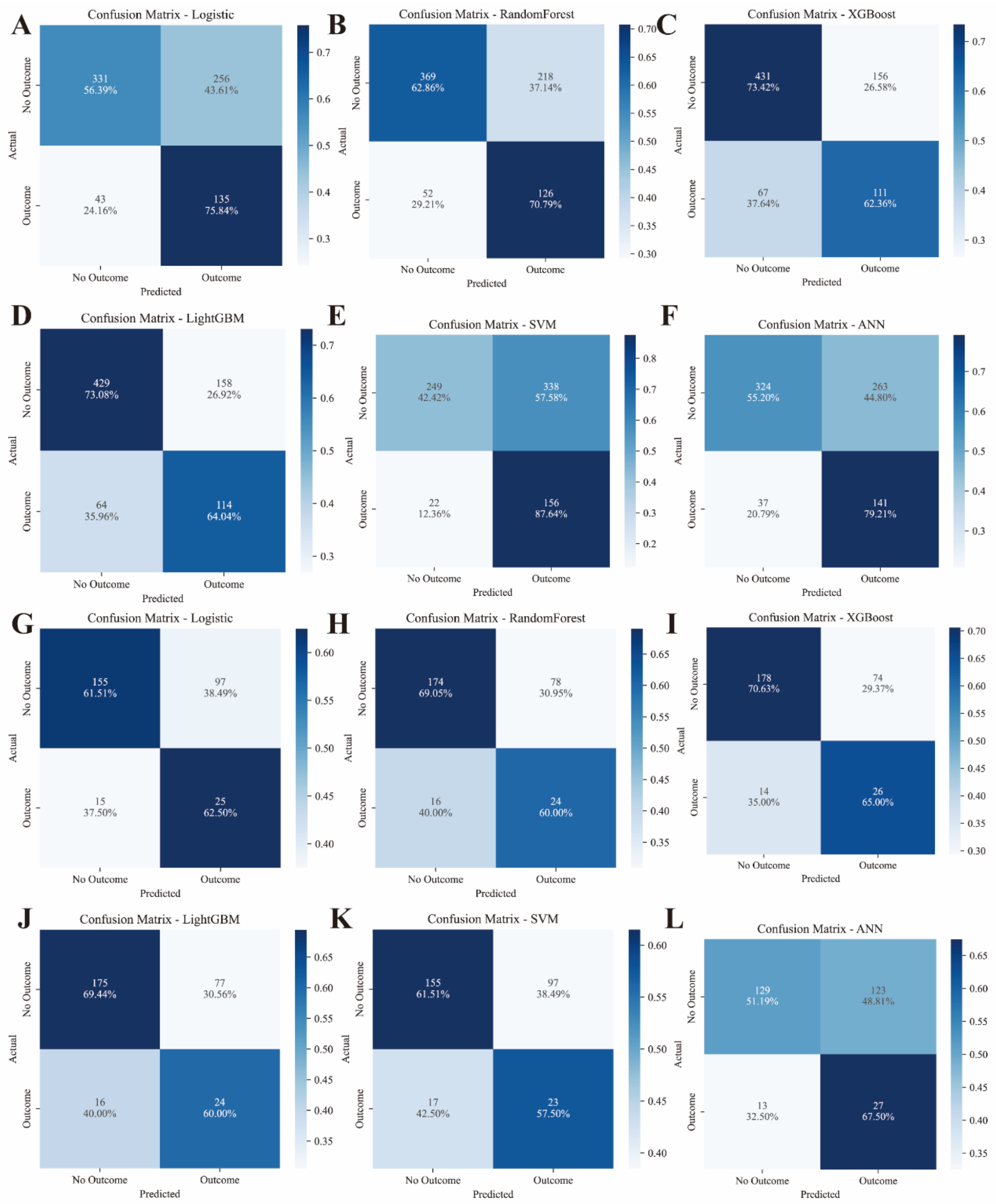

(A-F) Confusion matrix of internal validation set. **(A)** Logistic Regression (LR). **(B)** Random Forest (RF). **(C)** Extreme Gradient Boosting (XGBoost). **(D)** Light Gradient Boosting Machine (LightGBM). **(E)** Support Vector Machines (SVM). **(F)** Artificial Neural Network (ANN) **(G-L)** Confusion matrix of eICU CRD external validation set. **(G)** LR. **(H)** RF. **(I)** XGBoost. **(J)** LightGBM. **(K)** SVM. **(L)** ANN.

Table S2.

Performance of models in training set.

Models	AUC	AUC 95% CI Lower	AUC 95% CI Upper	Accuracy	Sensitivity	Specificity	F1 Score
LR	0.730	0.703	0.756	0.620	0.756	0.578	0.481
RF	0.938	0.925	0.949	0.709	0.971	0.630	0.610
XGBoost	0.840	0.819	0.862	0.674	0.868	0.615	0.555
LightGBM	0.907	0.890	0.923	0.659	0.945	0.571	0.564
SVM	0.718	0.691	0.745	0.611	0.722	0.577	0.465
ANN	0.730	0.703	0.756	0.651	0.715	0.632	0.489

LR, Logistic Regression; RF, Random Forest; XGBoost, Extreme Gradient Boosting; LightGBM, Light Gradient Boosting Machine; SVM, Support Vector Machines; ANN, Artificial Neural Network.

Figure S2.

Training set confusion matrix and model evaluation.

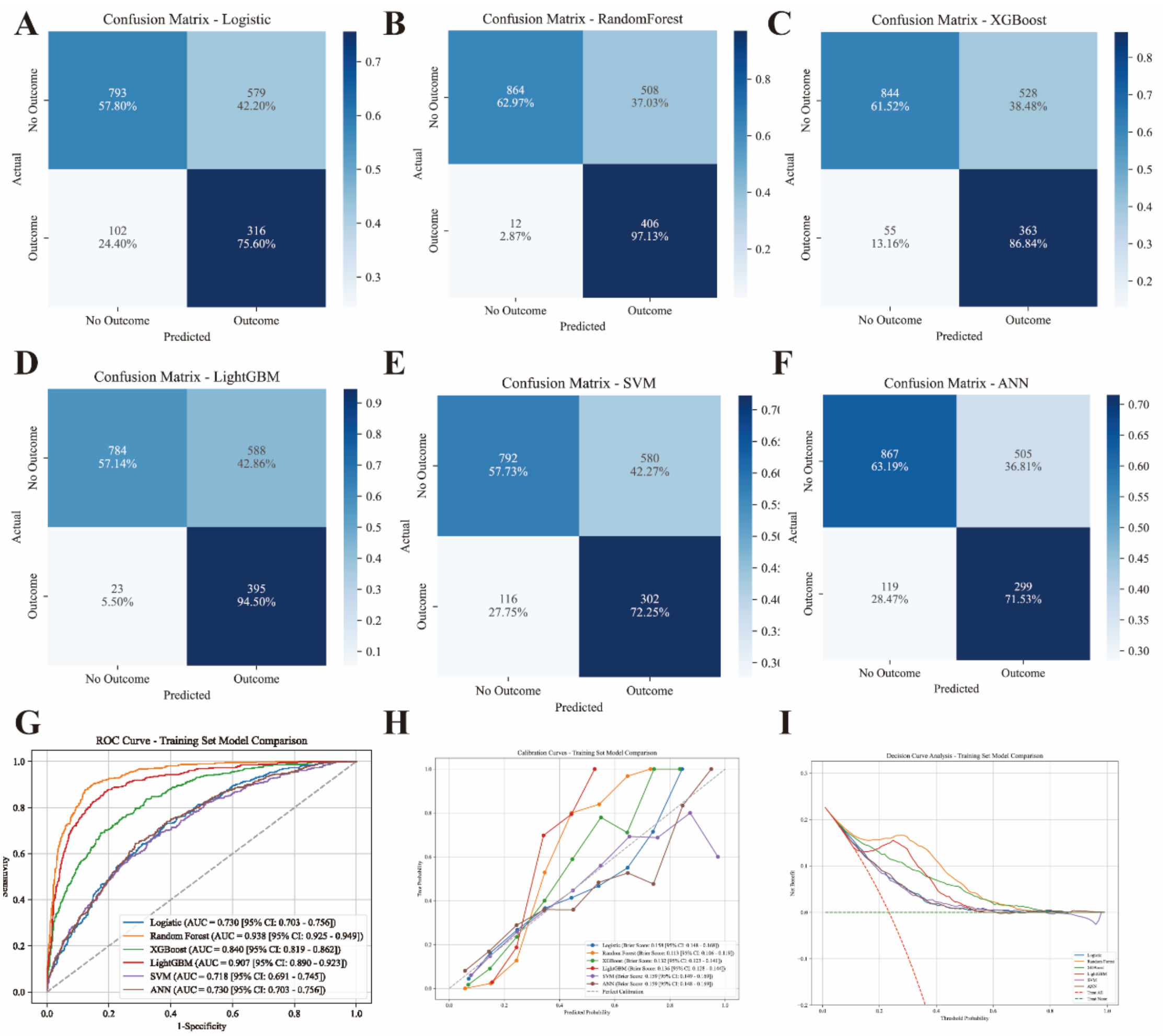

(A-E) Confusion matrix of training set. **(A)** Logistic Regression (LR). **(B)** Random Forest (RF). **(C)** Extreme Gradient Boosting (XGBoost). **(D)** Light Gradient Boosting Machine (LightGBM). **(E)** Support Vector Machines (SVM). **(F)** Artificial Neural Network (ANN). **(G-I)** Training set model evaluation. **(G)** Receiver Operating Characteristic (ROC). **(H)** Calibration curve. **(I)** Decision Curve Analysis (DCA).

Table S3.

Performance of models in external validation set.

Models	AUC	AUC 95% CI Lower	AUC 95% CI Upper	Accuracy	Sensitivity	Specificity	F1 Score
LR	0.736	0.640	0.823	0.771	0.500	0.813	0.374
RF	0.800	0.728	0.865	0.592	0.825	0.556	0.357
XGBoost	0.796	0.730	0.856	0.688	0.775	0.675	0.405
LightGBM	0.787	0.707	0.857	0.599	0.850	0.560	0.368
SVM	0.734	0.627	0.828	0.757	0.575	0.786	0.393
ANN	0.675	0.557	0.770	0.760	0.500	0.802	0.364

LR, Logistic Regression; RF, Random Forest; XGBoost, Extreme Gradient Boosting; LightGBM, Light Gradient Boosting Machine; SVM, Support Vector Machines; ANN, Artificial Neural Network.

Appendix S1.

28-day mortality rate of external validation set.

The external validation cohort included 292 patients, of whom 40 died within 28 days (13.7%).

Appendix S2.

Platform Description.

Our software platform has been published on GitHub: https://icu-risk-prediction-gyqyzthnshgukv8fpqgu9a.streamlit.app/. Users can access the latest version and updates of the application on this platform. Users can input demographic information, as well as vital signs, laboratory tests, medical history, etc., and then click the "run analysis" button to assess the early mortality risk of patients with COPD complicated by PH in ICU.

The prediction results primarily consist of two components:

(1) Early Mortality Risk Probability: Based on the input data, the prediction results will provide risk probability percentages.

(2) Feature importance analysis: Analyze the importance of each input parameter, and display the contribution of each feature to the prediction results through force plot and waterfall plot, helping users understand which factors have the greatest impact.

Table S4.

TRIPOD reporting checklist.

Item	Checklist item	Reported on page
**TITLE**		
**1 Title**	Identify the study as developing or evaluating the performance of a multivariable prediction model, the target population, and the outcome to be predicted.	p. 1
**ABSTRACT**		
**2 Abstract**	See TRIPOD for Abstracts checklist.	p. 2
**INTRODUCTION**		
**3a Background**	Explain the healthcare context (including whether diagnostic or prognostic) and rationale for developing or evaluating the prediction model, including references to existing models.	p. 3
**3b Background**	Describe the target population and the intended purpose of the prediction model in the context of the care pathway, including its intended users (e.g., healthcare professionals, patients, public).	p. 3
**3c Background**	Describe any known health inequalities between sociodemographic groups.	p. 3
**4 Objectives**	Specify the study objectives, including whether the study describes the development or validation of a prediction model (or both).	p. 4
**METHODS**		
**5a Data**	Describe the sources of data separately for the development and evaluation datasets (e.g., randomised trial, cohort, routine care or registry data), the rationale for using these data, and representativeness of the data.	p. 4

Item	Checklist item	Reported on page
**5b Data**	Specify the dates of the collected participant data, including start and end of participant accrual; and, if applicable, end of follow-up.	p. 4
**6a Participants**	Specify key elements of the study setting (e.g., primary care, secondary care, general population), including the number and location of centres.	p. 4
**6b Participants**	Describe the eligibility criteria for study participants.	p. 5
**6c Participants**	Give details of any treatments received, and how they were handled during model development or evaluation, if relevant.	No treatments received
**7 Data preparation**	Describe any data pre-processing and quality checking, including whether this was similar across relevant sociodemographic groups.	p. 5
**8a Outcome**	Clearly define the outcome that is being predicted and the time horizon, including how and when assessed, the rationale for choosing this outcome, and whether the method of outcome assessment is consistent across sociodemographic groups.。	p. 1-2, 7
**8b Outcome**	If outcome assessment requires subjective interpretation, describe the qualifications and demographic characteristics of the outcome assessors.	No need for subjective interpretation
**8c Outcome**	Report any actions to blind assessment of the outcome to be predicted.	p. 5
**9a Predictors**	Describe the choice of initial predictors (e.g., literature, previous models, all available predictors) and any pre-selection of predictors before model building.	p. 5

Item	Checklist item	Reported on page
**9b Predictors**	Clearly define all predictors, including how and when they were measured (and any actions to blind assessment of predictors for the outcome and other predictors).	p. 5
**9c Predictors**	If predictor measurement requires subjective interpretation, describe the qualifications and demographic characteristics of the predictor assessors.	No need for subjective interpretation
**10 Sample size**	Explain how the study size was arrived at (separately for development and evaluation), and justify that the study size was sufficient to answer the research question. Include details of any sample size calculation.	p. 4, 7
**11 Missing data**	Describe how missing data were handled. Provide reasons for omitting any data.	p. 5
**12a Analytical methods**	Describe how the data were used (e.g., for development and evaluation of model performance) in the analysis, including whether the data were partitioned, considering any sample size requirements.	p. 5
**12b Analytical methods**	Depending on the type of model, describe how predictors were handled in the analyses (functional form, rescaling, transformation, or any standardisation).	p. 15-17
**12c Analytical methods**	Specify the type of model, rationale, all model-building steps, including any hyperparameter tuning, and method for internal validation. Report this separately for all model-building approaches.	p. 6-7, 13-15; Supplementary Tables S1-S3

Item	Checklist item	Reported on page
**12d Analytical methods**	Describe if and how any heterogeneity in estimates of model parameter values and model performance was handled and quantified across clusters (e.g., hospitals, countries). See TRIPOD-Cluster for additional considerations.	p. 7
**12e Analytical methods**	Specify all measures and plots used (and their rationale) to evaluate model performance (e.g., discrimination, calibration, clinical utility) and, if relevant, to compare multiple models.	p. 11-14
**12f Analytical methods**	Describe any model updating (e.g., recalibration) arising from the model evaluation, either overall or for particular sociodemographic groups or settings.	Supplementary Appendix S2
**12g Analytical methods**	For model evaluation, describe how the model predictions were calculated (e.g., formula, code, object, application programming interface).	pp. 14-17
**13 Class imbalance**	If class imbalance methods were used, state why and how this was done, and any subsequent methods to recalibrate the model or the model predictions.	p. 5
**14 Fairness**	Describe any approaches that were used to address model fairness and their rationale.	Not applicable
**15 Model output**	Specify the output of the prediction model (e.g., probabilities, classification). Provide details and rationale for any classification and how the thresholds were identified.	pp. 17-19; Supplementary Appendix S2
**16 Training versus evaluation**	Identify any differences between the development and evaluation data in healthcare setting, eligibility criteria, outcome, and predictors.	p. 4, 7

Item	Checklist item	Reported on page
**17 Ethical approval**	Name the institutional research board or ethics committee that approved the study and describe the participant-informed consent or the ethics committee waiver of informed consent.	p. 23
**OPEN SCIENCE**		
**18a Funding**	Give the source of funding and the role of the funders for the present study.	p. 23
**18b Conflicts of interest**	Declare any conflicts of interest and financial disclosures for all authors.	p. 23
**18c Protocol**	Indicate where the study protocol can be accessed or state that a protocol was not prepared.	Public database
**18d Registration**	Provide registration information for the study, including register name and registration number, or state that the study was not registered.	Public database
**18e Data sharing**	Provide details of the availability of the study data.	p. 24
**18f Code sharing**	Provide details of the availability of the analytical code, including code for data cleaning, feature engineering, model building, and evaluation.	Supplementary Appendix S2
**PATIENT AND PUBLIC INVOLVEMENT**		
**19 Patient and public involvement**	Provide details of any patient and public involvement during the design, conduct, reporting, interpretation, or dissemination of the study, or state that there was no involvement.	p. 23
**RESULTS**		

Item	Checklist item	Reported on page
**20a Participants**	Describe the flow of participants through the study, including the number of participants with and without the outcome and, if applicable, a summary of the follow-up time. A diagram may be helpful.	p. 7; Figure 1
**20b Participants**	Report the characteristics overall and, where applicable, for each data source or setting, including the key dates, key predictors (including demographics), treatments received, sample size, number of outcome events, follow-up time, and amount of missing data. A table may be helpful. Report any differences across key demographic groups.	p. 7-10; Table 1
**20c Participants**	For model evaluation, show a comparison with the development data of the distribution of important predictors (demographics, predictors, and outcome).	p. 7-10; Table 1
**21 Model development**	Specify the number of participants and outcome events in each analysis (e.g., for model development, hyperparameter tuning, model evaluation).	p. 7-10; Supplementary Table S1
**22 Model specification**	Provide details of the full prediction model (e.g., formula, code, object, application programming interface) to allow predictions in new individuals and to enable third-party evaluation and implementation, including any restrictions to access or re-use.	Supplementary Table S1; Appendix S2
**23a Model performance**	Report model performance estimates with confidence intervals, including for any key subgroups (e.g., sociodemographic). Consider plots to aid presentation.	p. 11-14; Table 2; Supplementary Table S3

Item	Checklist item	Reported on page
**23b Model performance**	If examined, report results of any heterogeneity in model performance across clusters. See TRIPOD-Cluster for additional details.	p. 20
**24 Model updating**	Report the results from any model updating, including the updated model and subsequent performance.	Supplementary Appendix S2
**DISCUSSION**		
**25 Interpretation**	Give an overall interpretation of the main results, including issues of fairness in the context of the objectives and previous studies.	p. 20-22
**26 Limitations**	Discuss any limitations of the study (such as a non-representative sample, sample size, overfitting, missing data) and their effects on any biases, statistical uncertainty, and generalizability.	p. 22
**27a Usability of the model in the context of current care**	Describe how poor-quality or unavailable input data (e.g., predictor values) should be assessed and handled when implementing the prediction model.	p. 5
**27b Usability of the model in the context of current care**	Specify whether users will be required to interact in the handling of the input data or use of the model, and what level of expertise is required of users.	p. 18-19; Supplementary Appendix S2
**27c Usability of the model in the context of current care**	Discuss any next steps for future research, with a specific view to applicability and generalizability of the model.	p. 22

Table S5

Calibration of the external validation set.

**Metric**	**Estimate**	**95% CI**
Brier score	0.106	0.089 to 0.124
Calibration-in-the-large	−0.592	-0.955 to -0.305
Calibration slope	1.913	1.406 to 2.623
